# The Emergence of Psychoanalytic Metaneuropsychology: A Neuropsychoanalytically Informed Reconsideration of Early Psychic Development

**DOI:** 10.3389/fpsyg.2021.701637

**Published:** 2021-09-03

**Authors:** Matthew John Mellor

**Affiliations:** Department of Psychology, University of Exeter, Exeter, United Kingdom

**Keywords:** psychoanalysis, neuropsychoanalysis, controversial discussions, infant development, unconscious phantasy, object relations, ego development

## Abstract

This paper is principally concerned with reappraising some of the major disagreements that separated the Viennese and the London Kleinians during the British Psychoanalytical Society's Controversial Discussions. Of particular focus are questions pertaining to the genesis of ego development, the beginnings of object-relating, and the role of unconscious phantasy in respect of these phenomena. The aim of the investigation is to inquire into the light that may be shed on the once intractable conflicts surrounding these questions by bringing to bear more recent developments from psychoanalysis and the neurosciences. First, various key issues from the Controversial Discussions are outlined, before the paper turns to work by Jaak Panksepp and Mark Solms that bears on these older arguments and the Freudian theories that underpinned them. With these conceptual foundations established, three questions are posed and discussed with a view to understanding the implications of recent neuropsychoanalytic thinking for some of the entrenched conflicts that divided the British Society. These questions include: (1) what does it mean for the ego if the id is conscious? (2) What does recent neuroscientific knowledge tell us about whether the ego should be thought of as present from birth? (3) How can we understand and locate unconscious phantasy if the main part of the mind that Freud thought of as unconscious is not so? Research from the arena of infant development—particularly the material and analysis of infant observation—is drawn on to illustrate various conclusions. The paper ultimately concludes that taking such an interdisciplinary approach can reveal renewed justification for aspects of the Kleinian metapsychology.

## Introduction: An Uncivil War^1^

Between 1942 and 1944, in the wake of Sigmund Freud's death and with the world at war for the second time that century, the British Psychoanalytical Society was engaged in what is widely regarded as the most attritional debate in its history (Rycroft, 1968, p. 27). Dubbed the “Controversial Discussions,” the period played host to series of scientific meetings that were organised in the attempt to resolve conflicting views that different Society members held with respect to fundamental aspects of the psychoanalytic model of the mind. On one side of the divide stood the Viennese, comprised of Anna Freud and her followers, whilst on the other was a group of psychoanalysts loyal to Melanie Klein (Rycroft, 1968, p. 28). Despite the Controversial Discussions ultimately resulting in what may most appropriately be described as an agreement to disagree, their impact would shape British psychoanalysis for decades to come. Not only did the Discussions lead to the creation of a hitherto undefined “Middle Group,” they also necessitated significant changes to the Society's training programme which was reorganised to reflect the theoretical and technical divisions that had proved impossible to reconcile (King and Steiner, 1991, p. 25).

An important locus of the dispute between the Viennese and the Kleinians centred on questions pertaining to “the nature and function of phantasy” (Isaacs, 1948, p. 73). As stated by King and Steiner (1991, p. 242)—authors of the definitive account of the Controversial Discussions—“the notion of unconscious phantasy […] is probably the major theoretical theme of all the Scientific Discussions.” The different positions taken on this subject revealed deep divides within the British Society and led to radically divergent conclusions being drawn with respect to a broad range of psychological phenomena. A chief area of contention that ran parallel to the different conceptions of phantasy concerned the genesis of ego development. It was specifically on the question of whether the ego is present from birth that some of the Discussions' most stubborn disagreements were centred.

It is with these same themes that this paper is concerned. The aim of this investigation will be to inquire into how these once intractable conflicts may be reappraised by bringing to bear more recent developments from within psychoanalysis as well as from the neurosciences; an interdisciplinary emphasis that is closely compatible with the *Frontiers* Research Topic for which this paper has been produced. An outline of some of the key issues from the Controversial Discussions will be presented, before a review of theoretical literature by Jaak Panksepp and Mark Solms is addressed; work that influences contemporary understandings of these older arguments and the Freudian theories that underpinned them. This review of current interdisciplinary developments represents a key purpose of the present paper. With these conceptual foundations established, three questions are posed and discussed. The aim of this second half of the paper is to explore the implications of recent developments from the neuropsychoanalytic domain for some of the entrenched conflicts that divided the different cohorts within the British Society. Infant development research—particularly material and analysis derived from infant observation—is drawn on to give body to various conclusions.

## Controversial Discussions Concerning the Beginnings of Life

For the Viennese, it was fundamentally incorrect to suggest that the ego—the intrapsychic agency that Freud (1914, p. 7) had first described as “das Ich”– is present from birth. Claiming allegiance to Freud's original theories, Anna Freud proposed that at the very beginning of life “no ego exists” (King and Steiner, 1991, p. 420). Instead, the infant is said to be born into a state of “primary narcissism” that is characterised, in the Viennese view, as an “auto-erotic” phase that extends over the first months of life. Crucially, during this time, the infant is described as having “*no object relation* in the proper sense” (King and Steiner, 1991, p. 753–754, my emphasis). Rather, it is argued that the infant spends their opening months seeking only “*themselves* as a love-object,” to quote Sigmund Freud's paper *On Narcissism* (1914, p. 88). Moreover, as Freud (1914, p. 100) would go on to state in that influential paper, “the development of the ego consists in a departure from primary narcissism and gives rise to a vigorous attempt to recover that state.” In other words—and as was at the very crux of the arguments put forward by Anna Freud and her followers—a basic mutual exclusivity exists between the presence of primary narcissism and the existence of an ego that can relate to objects “in the proper sense,” such that the latter is conditional on the suspension of the former.

For the Kleinians, this perspective constituted an essential misunderstanding of the infantile psyche. What's more, rather than following Freud, it was contended that the Viennese Group's proposals in fact amounted to “a distortion” of his views (King and Steiner, 1991, p. 244). In contrast to Anna Freud's position, the Kleinians affirmed that the ego *does exist* from birth; indeed, they suggested that its “considerable capacities” can be observed from the very start of life (Bott Spillius et al., 2011, p. 319). Amongst these capacities is said to be “a pre-existing knowledge of the breast and of the mother” on which and whom the child depends (Bott Spillius et al., 2011, p. 319–320). In the Kleinian schema, the expression of this inherited relational knowledge is *mediated by phantasy*, which in Thomas Ogden's terse description, can be defined as the “psychic representation of instinct” (Ogden, 1984, p. 501). Accordingly, phantasy—the psychological “corollary” of our innate biological instincts—is conceptualised as accompanying all mental activity from the very beginning of life (Bott Spillius et al., 2011, p. 3–4). In the view of Klein herself, as the ego-equipped infant navigates its primordial experience and forges burgeoning relationships with its primary objects, its phantasy is said to be governed by the primal mechanisms of “projection and introjection” (Bott Spillius et al., 2011, p. 320). The resultant flow of psychical qualities and quantities that these mechanisms engender represents mental activity that, through the Kleinian lens, co-occurs with the rudimentary ego's oscillation “between states of integration and disintegration” (Klein, 1946, p. 179–180). Consequently, phantasy and ego are regarded as intimately connected: specifically, the presence of phantasy is understood as having a cognate co-existence with “das Ich.”

With the establishment of these basic theoretical tenets, it became impossible for the Kleinians to maintain that a phase of proper primary narcissism and auto-erotism exists in the baby during the first months of life (King and Steiner, 1991, p. 253). To be clear, it was not that the Kleinians saw narcissism per se as an invalid concept. Rather, they argued that narcissism represents a defensive withdrawal to an idealised internal object and thus can only be conceived of as *secondary* (King and Steiner, 1991, p. 253). For the Viennese, it proved similarly impossible to “accept the notion of unconscious phantasy” in regard to the infant's first days, as to do so would be to imply the existence an early ego (King and Steiner, 1991, p. 243). For those that participated in the Discussions, this impasse was largely irreconcilable. Perhaps the closest that the two groups came to agreement is evidenced in Anna Freud's admission that the “synthetic function of the ego”—by which she is referring to “the synthesis of perception and reality-testing”—is “achieved in degrees from birth onwards” (King and Steiner, 1991, p. 420). Nonetheless, she would go on to state that “it would not be accurate to say that this is achieved by the ego,” but rather that this “synthetic function builds the ego or constitutes *the first ego-nucleus*” (King and Steiner, 1991, p. 420, my emphasis). In Anna Freud's consistent view, it was not until “six months of age” that an ego-nucleus could be said to have developed into an ego structure capable of object relating (King and Steiner, 1991, p. 754).

## Panksepp's Contribution From an Adjacent Domain

In the middle of these Controversial Discussions—in June 1943—a child was born in Estonia, who, later in life, would transform our understanding of the emotions through a string of neuroscientific discoveries. Named Jaak Panksepp, the discoveries he made can arguably make a profound contribution to our retrospective appraisal of the disagreements that divided the British Psychoanalytical Society at the time of his birth. Given their import, these discoveries are worth briefly sketching out. At the centre of Panksepp's contribution lies his work on the basic emotion systems: “consciousness-creating affective circuits” that are “concentrated in subcortical regions” (Panksepp and Biven, 2012, p. 1). Taken together, these evolutionarily ancient subcortical regions of the brain comprise “at least seven emotional, or affective, systems”: “SEEKING (expectancy), FEAR (anxiety), LUST (sexual excitement), CARE (nurturance), PANIC/GRIEF (sadness), and PLAY (social joy)” (Panksepp and Biven, 2012, p. 2).” For Solms (2018, p. 5)—neuroscientist, psychoanalyst and long-standing collaborator of Panksepp's—*these* are “the drives” of traditional psychoanalysis, albeit re-imagined and refined. Crucially, unlike the homeostatic drives which compel us to seek, amongst other things, sleep, sustenance and thermal regulation, the basic emotion systems outlined here constitute *relational drives*, the satisfaction of which routinely depends on objects (in the psychoanalytic sense) (Solms, 2018, p. 5). In short, to feel the affects generated by these neurological systems is to experience a sense of how one's basic emotional needs may or may not be being met (Solms, 2018, p. 5). Indeed, that, for affective neuroscientists, is the primary function of feeling: to tell you “*how you are doing* in relation to your biological needs” (Solms, 2021, p. 96).

It is this array of subcortical systems that, according to Panksepp and Biven (2012, p. 13), creates an “energetic form of consciousness”—what is also referred to as “core consciousness” (Damasio, 2000, p. 82)—that is full of raw affective intensity. Put simply, it *feels* good or bad—pleasurable or unpleasurable—to experience the consciousness that these systems create (Solms, 2021, p. 96). This leads to a conclusion with radical implications, not least because it clearly not only applies to humans: for an organism to be feeling the status of its biological needs at any given moment necessarily implies the presence of core consciousness. As Panksepp dedicated much of his working life to investigating, such “affective consciousness” (Panksepp and Biven, 2012, p. 2) can be generated using deep brain stimulation techniques that target the seven basic systems (Panksepp, 2010, p. 536). Moreover, this experimental method can reliably produce expected behavioural responses. For example, an organism experiencing the activation of their SEEKING system will exhibit foraging behaviours; if the FEAR system is activated, a fright or flight response will predominate etc. (Panksepp, 2010, p. 536–537). And whilst it cannot be claimed that the seven basic emotions, on their own, encapsulate the extensive nuance of human feeling, they are said to underlie that vast richness. For Panksepp, this narrow range of systems represent the *primary* sources of our emotional life. The great variety of “secondary” emotions that we experience are generated, it is argued, through “complex cognitive-affective amalgams” (Panksepp and Watt, 2011, p. 10) of the basic emotions that lie at the “deepest roots of the human mind” (Panksepp and Biven, 2012, p. 5), analogous to the way in which the full spectrum of colour may be created through mixing various quantities of red, yellow, and blue.

## Solms' Proposed Changes to the Psychoanalytic Lens

Amongst the most established of psychoanalytic theories to have been shaken by the identification of the basic emotions relates to a framework that both the Viennese and the Kleinians stated their allegiance to: Freud's structural model of the mind. First detailed in the 1923 paper *The Ego and the Id*, the structural model represented Freud's proposal that the personality is comprised of three psychic agencies: the id, the superego, and as was so keenly debated during the Discussions, the ego. This structural model constituted an expansion of Freud's original model of the mind—the topographical model—which he had introduced in 1900 as a way of mapping the unconscious, preconscious, and conscious psychological domains (Boag, 2017). Importantly, the structural model of 1923 did not operate as a replacement for Freud's topographical account; rather, the two theoretical frameworks are more accurately viewed as describing different dimensions of the human mind, analogous to the way in which a body of water can be understood in distinct yet mutually inclusive ways: in one sense, we might consider its depth-related hydrostatic pressure; in another, we can attend to its molecular structure. Correspondingly, the components of Freud's structural model may be compatibly located within—and across—the various topographical layers of the psyche.

Of all the components within the structural model, it is perhaps Freud's conceptualisation of the id that has undergone the most dramatic reappraisal due to Panksepp's discoveries (Solms, 2013, p. 5). For Freud (1938, p. 145), the id was described as the part of the mind that contains “the instincts, which originate from the somatic organization.” As is unambiguously stated in *An Outline of Psychoanalysis*, “the id obeys the inexorable pleasure principle”: it is despotically governed by “feelings of pleasure-unpleasure” (Freud, 1938, p. 198).

As may be apparent, the Freudian id thus bears the some of the same core properties as Panksepp's basic emotion systems: both are described as the source of the instincts and both are governed by the pursuit of satisfying pleasure (Solms, 2013, p. 7). Where the similarity between the id and the basic emotions ends however is in the fact that the id is located by (Freud, 1923, p. 24) within the *system unconscious*. For affective neuroscientists, the basic emotion systems are understood in wholly opposite terms. Whilst these basic affects are also described as instinctually directing the organism through “feelings of pleasure-unpleasure,” they are far from being unconscious; indeed, they have been shown by Panksepp to be “the font of *all* consciousness” (Solms and Panksepp, 2012, p. 174), my emphasis). This conflict is only resolved, Solms and Panksepp (2012, p. 174) suggest, if we are willing to reconceptualise the Freudian id as *essentially conscious*. Not only does this challenge one of the bedrocks of psychoanalysis, it also upturns one of the theoretical foundations that the Viennese and the Kleinians agreed upon. Arguably however, rather than debunking the structural model entirely, Solms (2019) suggests that Freud may have simply got it, figuratively speaking, “upside-down,” a view that implies the model may still represent an invaluable organisation of psychological phenomena, notwithstanding the re-orientations that it may require.

## Towards A Biological Basis for Psychoanalytic Metapsychology

As affective neuroscientists have demonstrated over recent decades, the basic emotion systems that underpin Solms and Panksepp's recalibration of psychoanalytic metapsychology each have “a distinct brain anatomy, neuropharmacology, and physiology” (Davis and Montag, 2019, p. 2). For an encyclopaedic account of this detail, *The Archaeology of Mind* (Panksepp and Biven, 2012) represents an authoritative source. Whilst a full description of this biology and chemistry lies outside the scope of the present paper, a basic overview is nonetheless warranted. Indeed, one of the most significant implications of these discoveries relates to the brain's anatomy and how we understand the functional contributions of different brain regions. As Panksepp and Biven write:

“*the basic biological values of all mammalian brains were built upon the same basic plan, laid out in consciousness-creating affective circuits that are concentrated in subcortical regions, far below the neocortical ‘thinking cap' that is so highly developed in humans.”* (Panksepp and Biven, 2012, p. 1)

This statement is radical for numerous reasons. Not only does it invite a re-evaluation of man's place in nature and broach what has enigmatically been called “the final frontier” of science: *consciousness itself* (Gunamuktananda, 2014), it also poses a specific challenge to conventional wisdom within brain science and beyond. As Solms (2013, p. 9) notes, Panksepp's proposal exposes “the corticocentric fallacy”: the long-held notion that “the ‘seat' of consciousness is in the cerebral cortex,” to quote Freud who also ascribed to this view (Freud, quoted in Solms, 2013, p. 9). For Panksepp and colleagues however, decades of inquiry strongly suggest that “the seat” of consciousness is in fact situated, anatomically speaking, “*far below*” the cortical brain regions (Panksepp and Biven, 2012, p. 1).

Powerful evidence for this claim is provided from various sources, perhaps none of which are more compelling than the cases of hydranencephalic children documented by Merker (2007). As Davis and Montag (2019, p. 3) write, such children – who are born without a “neocortical ‘thinking cap',” and yet who are *highly emotional* in ways that are “situationally appropriate” – unambiguously show that emotional responses, even in humans, “do not require the participation of the neocortex.” If the cerebral cortex does not then play an integral role in the generation of emotion, which subcortical brain structures do? In Panksepp's (2010, p. 535) words:

*The primary-process networks for emotional instincts run from midbrain periaqueductal gray (PAG) regions to medial diencephalon to various basal ganglia nuclei (amygdala, bed nucleus of the stria terminalis, nucleus accumbens, etc.) that interact with paleocortical brain functions (e.g., cingulate, insular, as well as medial- and orbitofrontal cortices) and more indirectly with certain neocortical regions to provide integration with higher cognitive activities*.

Due to the “striking cross-species homologies” that exist with respect to the more ancient primary-process neural regions, affective neuroscientists have been able to study many of these regions in animal subjects as a means of deriving insights into the human brain (Panksepp, 2010, p. 534). A crucial discovery to have emerged from such investigations concerns the central role played by the periaqueductal grey (PAG): the structure that is the “terminus of every affect circuit” (Solms, 2020, p. 14), “the genesis of every newly felt affect” (Solms, 2020, p. 14), and that's “common” significance to all affective systems has been corroborated in meta-analyses (Buhle et al., 2013; Motta et al., 2017; Gammon, 2020, p. 198). As Solms writes in his 2021 volume, *The Hidden Spring*, when describing this structure:

“*[The PAG] divides into two groups of functional columns. One of them, the back one [dorsal] […] is where the FEAR, RAGE and PANIC/GRIEF systems terminate. The front one [lateral] [is where] […] the LUST, CARE and SEEKING circuits terminate*” (2021, p. 137).

As such, the PAG can be seen to play a vital part in generating the very feelings of pleasure and unpleasure that are outputted by the brain's consciousness-creating affective circuits and that define the re-conceptualised id. This said, as Kunstadt (2013, p. 56, my emphasis) observes, it would not be accurate to state therefore that “consciousness *resides* in the PAG” and that this structure alone represents the “neural correlate of consciousness” (Koch et al., 2016). Indeed, correlating consciousness in a localised manner with the PAG runs counter to many of the compelling arguments put forward by embodied cognition theorists who emphasise the importance of the whole body—not just the brain—in the biology of emotion (e.g., Antonio Damasio's Somatic Marker Hypothesis and Stephen Porges' Polyvagal Theory) (Hemp et al., 2014). There is, however, good evidence to suggest that when it comes to the networked generation consciousness, the PAG functions as a crucial “nodal point”: it is, as Solms (2013, p. 12) states, the “smallest region of brain tissue in which damage leads to total obliteration of consciousness,” (something that is *not* observed in the aforementioned cases of hyranencephaly). In the same sense, whilst it would not be meaningful to exclusively correlate the id with this localised brain structure, it could be argued that the PAG is centrally implicated in the mental functioning associated with the id.

## Behaviourist Criticism of the Interdisciplinary Project

Critiques of the views hitherto explored—particularly of psychoanalytic ideas—have been widely documented, both within and outwith the psychological and brain sciences. Perhaps the most influential and sustained criticism has been levelled by behavioural psychologists such as B.F. Skinner. Claimed by Haggbloom et al. (2002) to have been “the most eminent psychologist of the twentieth century,” Skinner was one of the founders of behaviourism, which, over the course of last century, came to dominate the discipline of psychology. A consistent position taken by Skinner (1950, p. 193) was to be critical of “any explanation of an observed fact which appeals to events taking place somewhere else, at some other level of observation, described in different terms, and measured, if at all, in different dimensions.” As Zilio (2016, p. 202) explains, a problematic theory would thus be one “that attempts to explain behaviour by describing events that are not part of the behavioural relation such as physiological and mental events.” Accordingly, both neuroscience and psychoanalysis have come in for criticism. For Skinner, the notion that expressed behaviour may be “explained” by detailing, for example, the functioning of the basic emotion systems, would be fundamentally flawed; because such phenomena occur “in different dimensions,” the “radical behaviourist” (Naour, 2009) view suggests that a different “level of observation” (Skinner, 1950, p. 193) is necessarily required.

Through applying this logic, Skinner argued that many within the psychological and brain sciences—including Freud himself—proposed the existence of an “identity relation” between conceptual models derived from the distinct phenomena of behaviour and neurological functioning, simply as a means of avoiding the “accusation of dualism” (the theory that the mental and the physical—or mind and brain—“are, in some sense, radically different kinds of thing” (Zilio, 2016, p. 204; Robinson, 2020). As Skinner wrote in 1983:

*A touch of physiology seems to save them [cognitive psychologists] from dualism, and many of them use ‘brain' and ‘mind' interchangeably. Freud took a similar position much earlier. He assumed that we should some day know what the ego, superego, and id, the conscious, preconscious, and unconscious, and all the dynamisms really were in neurological terms* (Skinner, 1983, p. 10).

The argument here is that by accepting such “identity relations” as valid, we grant ourselves carte blanche to propose hypothetical models of mental processes inferred from behaviour without any concern about validation and parsimony (Zilio, 2016, p. 204–205). To proceed in such a fashion would, it is alleged, be deeply unscientific.

Whilst it is indeed true that a core aspect of the neuro-psychoanalytic endeavour is to build conceptual bridges between the neurosciences and psychoanalysis, it is also true that this project fundamentally aims at establishing *more* scientifically robust levels of validation through “mutually enriching dialogue” (Yovell et al., 2015), not less. The chief goal of neuropsychoanalysis is, after all, to facilitate the *triangulation* of discoveries made within interfacing disciplines that all pertain, as Solms (2014) states, to “the same part of nature.” By the same token, such triangulation can enable disproven hypotheses to be rejected; a vital cornerstone of the objective scientific method. This very ambition is what underpins the present paper. The principal research question for this conceptual analysis is thus: through engaging in an interdisciplinary dialogue and learning lessons from contemporary neuroscience, what re-envisioned shape does psychoanalytic metapsychology take? How, to borrow a term from anthropology, can we arrive at a “thick description” (Geertz, 1973, p. 3) of the psyche by expanding our horizons and incorporating new neuroscientific dimensions to develop a *meta-neuro-psychological* “way of seeing” (Berger, 1972)? Specifically, which of the explanations put forward by the Viennese and the Kleinians during the Controversial Discussions may now be regarded as having the more solid scientific justification?

## Discussion: Emergent Questions

Innumerable questions emerge from Solms' neuropsychoanalytic re-orientation of Freud's structural model. To cite but a few of them: what does it mean for the ego if the id is conscious? What does recent neuroscientific knowledge tell us about whether the ego should be thought of as present from birth? And not least, how can we understand and locate unconscious phantasy if the main part of the mind that Freud thought of as unconscious is not so? There are, of course, many more questions that arise in addition to these, however for the purposes and scope of this paper, it is these that will be taken in turn.

### What Does It Mean for the Ego if the Id Is Conscious?

As for the first of these questions, a similarly radical revaluation of traditional psychoanalytic perspectives may be warranted. For Freud (1923, p. 17–23), the ego was defined as “the coherent organization of mental processes” present in each individual that “seeks to bring the influence of the external world to bear upon the id” and its incomparably passionate tendencies. Put succinctly, it is the mental representative of external reality (Freud, 1923, p. 36). Crucially, this representative function inextricably links the ego with the body: the flesh and bone that occupies time and space as an object *in external reality*. As Freud (1923, p. 26) memorably encapsulated it, “the ego is first and foremost a bodily ego; it is not merely a surface entity, but is itself the projection of a surface.” Correspondingly, the ego may thus be conceived of primarily as the body's *re-presentation* within the mind. Moreover—and central to this discussion—the ego was thought by Freud (1923, p. 17–19) to have an important (although not exclusive) relationship to consciousness. As he writes in *The Ego and the Id* when first introducing the “mental agency” of “das Ich,” “it is to this ego that consciousness is attached” (Freud, 1923, p. 17–19). And whilst it is important to acknowledge that, in Freud's (1923, p. 19–24) view, parts of the ego—for example “the repressed” aspects— “can be unconscious,” it was *only* the id that was described by him as essentially and exclusively “unknown and unconscious.” By contrast, the ego, more so than any of the other psychic agencies that entered psychoanalytic parlance in 1923, was what Freud linked with consciousness.

Returning to the neuropsychoanalytic notion that the structural model may benefit from being upended, the implications for the ego are perhaps predictable. According to Solms and Panksepp (2012 p. 174, my emphasis) the mental functioning that is considered synonymous with the Freudian ego is, contrary to the conventional view, “*unconscious in itself*.” The variety of mental functioning being referred to here is what Solms (2013, p. 16), in his paper *The Conscious Id*, explicitly links with “the external self”: the “learnt representation” of the body within the mind. For Solms (2013, p. 16), this ideational “external self”—this ego—is intimately connected to the perceptual and representational level of experience that confers a sense of our existence as an object amongst objects. The ego is thus said to enable the ability to go beyond *just feeling* (as is synonymous with the Freudian id), to “feeling this *about that”* (where “*that*” is an object of perception) (Solms and Panksepp, 2012, p. 168; Solms, 2013, p. 16).

This contextualising objectification of feeling goes to the very heart of the ego's relationship with the id: the former is said to stabilise the latter's core consciousness by “transforming affects into object representations” (Solms and Panksepp, 2012, p. 174). A crucial dynamic to note here is that this transformation process is *galvanised by affect*. Without an affective stimulus from the id, the ego would have nothing to declare and therein could not be described as conscious. It is for this reason that the ego is argued to be “unconscious in itself”; the ego relies on the output of the id's affective circuits “from their origin in some of the most ancient strata of the brain” for its irrigation and so that the “dead soil” of its unconscious representations may be brought “to mental life” (Solms, 2021, p. 91). Incomplete accounts of this subtle but important distinction are arguably why consciousness has frequently been associated with the ego, not least by Freud himself.

For Solms and Panksepp (2012, p. 173), the level of experience conferred by the ego—i.e., the sense of self as an object that “feels this *about that*”—is described as “second-person perspective.” Such a perspective can be conceptually located between a “lower” viscerally affective “first-person perspective” that *just feels* (as has been considered as closely related to a conscious id), and a “higher” re-representational “third-person perspective” that enables the reflexive capacity to perceive the self from an external perspective (Solms and Panksepp, 2012, p. 173–174; Solms, 2013, p. 16). This “multi-tiered” framework of primary, secondary and tertiary levels of experience is, for Solms and Panksepp (2012, p. 145) a structural parsing of consciousness that closely echoes the philosophy of Endel Tulving. In Tulving's (1985, p. 1) view, consciousness proceeds from the “lowest” *anoetic* level, through the *noetic* level, to the “highest” *autonoetic* level. Put briefly—and in a manner that aligns with Solms and Panksepp's (2012, p. 145–146) tripartite structure—*anoetic* consciousness refers to unthinking forms of experience which may be affectively intense without being “known”; *noetic* experience is linked to exteroceptive perception and cognition; *autonoetic* experience refers to abstracted forms of perceptions and cognitions, which facilitate conscious “awareness” and reflection.

As Panksepp's work has shown, anoetic consciousness is something that evidence suggests is experienced across much of the animal kingdom (Panksepp and Biven, 2012, p. 1). By contrast, autonoetic consciousness is a much rarer phenomenon that, even within humans, is not said to be exhibited “before the age of four” (Vandekerckhove, 2009, p. 9). This brings us to a crucial point towards which this paper has been converging: the different theoretical positions in respect of the ego that the Viennese and the Kleinians espoused during the Controversial Discussions may be seen to relate to *different levels of consciousness*. In the Kleinian view, a “lower” point in Tulving's hierarchy would indicate early ego functioning. For Anna Freud and her followers, something categorically more advanced in this hierarchy would be necessary to claim the existence of an ego. Crucially, if we accept Freud's (1923, p. 36) notion that “the ego is essentially the representative of the external world,” then a level of consciousness that incorporates the capacity for exteroceptive perception is a pre-requisite for the ego's existence. Accordingly, the question about the genesis of ego-presence might thus be recast as a question about the point at which a nascent second-person perspective, however rudimentary, first introduces the object-relational capacity to “feel this *about that*.”

### What Does Recent Neuroscientific Knowledge Tell Us About Whether the Ego Should Be Thought of as Present From Birth?

In their 2012 paper *The Id Knows More Than the Ego Admits*, Solms and Panksepp elaborate on the second Tulvingian level of consciousness—the *noetic* level—and introduce a further concept that appears to equate this with a type of memory which has a specific aetiology that can be dated to certain points in infancy. The type of memory introduced is that of the “declarative” category; they write “the ‘declarative' noetic self” is what can be regarded as “synonymous with Freud's ‘ego”' and “unconscious in itself” (Solms and Panksepp, 2012, p. 174). It is worth unpacking the implications of this equivalence as they bear on the disagreements of the Controversial Discussions with some significance.

Declarative memory is defined as a type of long-term memory (as opposed to short-term “working memory”) that “requires conscious recollection and includes the recognition and recall of names, objects, and events” (Bauer and Pathman, 2020, p. 1). In the literature, it is interchangeably referred to as “declarative” and “explicit” memory. Recent research investigating infant behaviour on non-verbal, imitation-based tasks has shown that this type of memory *is* apparent in the first year of life; specifically, even 6-month-olds appear able to remember actions for 24 (but not 48) hours (Bauer and Pathman, 2020, p. 2). During the months that follow, this recall timeframe expands exponentially: 9-month-olds appear to be able to remember actions for 1 month, and by 20 months of age, infants can remember for as long as 1 year (Bauer and Pathman, 2020, p. 1). If therefore it is appropriate to associate declarative memory with ego function, it would suggest that the Viennese view whereby the ego is not present until “6 months of age” (King and Steiner, 1991, p. 754) may represent the more accurate account of infant development.

However, as has been shown by cognitive neuroscientists, declarative memory is not the only type of long-term memory that humans are equipped with: non-declarative (or “implicit”) memory represents another form. This second type of long-term memory is defined as inaccessible to conscious awareness and includes “skill learning,” “emotional learning,” and “priming” (i.e., facilitated processing of a stimulus—such as a mother's face—as a function of prior experience with it) (Bauer and Pathman, 2020, p. 1; Squire and Dede, 2015, p. 3). Crucially, the latest infant development research indicates that non-declarative memory is apparent “*virtually from birth,”* a finding evidenced by how infants show more robust processing of faces they have perceived before relative to novel faces (Bauer and Pathman, 2020, p. 1, my emphasis). If it is legitimate to entertain a broader classification of mnemonic function as indicative of the ego—specifically if the operation of declarative memory *and* non-declarative memory can both be conceptualised as ego functions (albeit involving different levels of sophistication)—then the Kleinian position is given significant credence.

Interestingly, close inspection of two of Solms' papers referenced above—*The Id Knows More Than the Ego Admits* (2012) (co-authored with Panksepp), and *The Conscious Id* (2013), which represents a “substantially revised version” (2012, p. 143) of the 2012 paper—reveals that reference to the “declarative self” undergoes conspicuous amendment; usage of this term when stating the “major conclusion” in respect of the ego's unconscious status, whilst present in 2012, is dropped in the 2013 paper in favour of “the external self” (Solms and Panksepp, 2012, p. 174; Solms, 2013, p. 16). One explanation for this subtle shift might be that the circumscribed equation of the ego with “the declarative self” and its associated memory capacity was deemed too limiting. Indeed, such a reappraisal may have been warranted as the original wording implies the following logic:

a) if the declarative self which does not appear to develop before 6 months of age is exclusively concomitant with the ego (as per a neo-Viennese view), and;
b) the presence of an ego co-occurs with object-relating (as both the Viennese and the Kleinians maintained during the Controversial Discussions), then;
c) the infant is necessarily *not* an egoic object-relating entity at birth.

However, such a conclusion is arguably not borne out by the research. As alluded to above, new-borns *can and do* exhibit non-declarative priming capacities; they will “show more robust processing” of their mother's face than they do a stranger's. If such perceptually-driven priming can be regarded as evidence of object-relating—a position that arguably has greater logical coherence than to suggest such behaviour implies auto-eroticism—then the workings of rudimentary non-declarative memory may be conceived of as conferring a nascent second-person perspective, and therein *egoic experience* of the most embryonic kind.

#### The Clinical Material of Infant Observation

Given the developmental period under consideration here, infant observation data constitutes a natural source of insight. Moreover, as has been noted by Rustin (2010, p. 382), psychoanalytic infant observation can be seen to “preserve several of the attributes of the clinical setting” and, as such, can be considered a forum for the development of “psychoanalytic theory and technique,” akin to the traditional consulting room. A recent contribution to this area of knowledge comes from a student of Rustin—Wendy Shallcross—who undertook “the systematic study of a single recorded case of infant observation using Grounded Theory” (2014, p. 1). As Rustin (2016, p. 188) points out, himself referencing Anderson (2006), the Grounded Theory methodology is “well-suited” to infant observation-based research “because its analytic procedures are so close to the ‘line by line' practise of supervision through which they [child psychotherapists] have previously learned to reflect on the meanings of clinical material”). It is to this fine-grain analysis of infant observation that we shall now turn.

Shallcross (2014, p. 28) begins her thesis with an acknowledgment of the importance placed on attempting to suspend “possible theoretical explanation or theorization until analysis of the observational material was complete.” She then goes on to depict, both via the raw observational data and its ultimate analysis, the remarkable sensitivity that the infant shows towards their environment whilst awake as well as, strikingly, *whilst asleep*. Indeed, one of the most notable features of the recorded observations is how the baby, Kieran, “although sleeping, registers the to-ing and fro-ing of his mother” (Shallcross, 2014, p. 74). The following is reported of the second observation, at which point Kieran was only 10 days old:

*The pram was situated opposite the window, diffused light shone onto Kieran's face. He lay with his head to the right, lying predominantly on his back. Kieran shuddered as his mother left the room. A smile flickered across his face, followed by a sucking movement and irregular breathing. Kieran's eyelids continued to flutter and occasionally became screwed quite tightly, at such times he looked as though he may be in pain or remembering pain. Susan returned, she looked into the pram and watched Kieran for a short while. Kieran's breathing steadied into a slower rhythm, his body still and relaxed*. (Shallcross, 2014, p. 74)

Whilst this brief excerpt does not constitute conclusive evidence on its own, Kieran's sensitivity to his mother's presence is representative of his presentation throughout the second observation and is echoed across many of the 46 observations that form the basis of Shallcross' research. What's more and as the author herself notes, to have detected this “mental relatedness” at such an early stage and without the infant even occupying a waking state represents an “original finding” of some import (Shallcross, 2014, p. 34).

Further to the study's raw data, Kieran's “mental relatedness” constitutes a discernible feature of the data analysis. This is recognisable within many of the codes that emerged through the initial line-by-line coding of written reports and is implicit in a variety of the overarching thematic clusters that were identified through the subsequent grouping of these codes (Shallcross, 2014, p. 204–211).^2^ Clustered themes that emerged through analysis of the initial four observations from the first month of Kieran's life notably include “Immersion” (in the object), “Orientation” (to the object), and “Transformation” (through the object) (Shallcross, 2014, p. 204–211). A distinct and compelling object relational resonance was thus found to exist across much of the observational data, including that relating to Kieran's very earliest days. For Shallcross (2014, p. 28), this apparent object relating—which, as has been discussed, carries profound implications for the early ego—was primarily expressed through the infant's body; as she writes, “there was observable bodily organisation and rhythm in the baby's movements, particularly so in the presence of mother, which I came to realise indicated a significant level of integration.” Consequently, having aimed to suspend premature theorising, Shallcross (2014, p. 28) ultimately concludes that her “findings are consistent with Melanie Klein's account of how object relations are operative from birth.”

### How Can We Understand and Locate Unconscious Phantasy if the Main Part of the Mind That Freud Thought of as Unconscious Is Not so?

We now return to where this paper began: “the nature and function of phantasy” (Isaacs, 1948, p. 73). However, we can now bring to bear on that central theme of the Controversial Discussions the developments detailed in this paper. As was alluded to in the opening sections, phantasy—that which is defined by Kleinians as “the psychological corollary of instinct”—has a cognate co-existence with the ego such that it is not possible to conceptualise the presence of one without implying the existence of the other (Bott Spillius et al., 2011, p. 3–4). The logic underpinning this is that it is through the *medium of phantasy* that affects (understood here as instinctual expressions of the basic emotion systems) are transformed into object representations: the hallmark of ego function. As such, affect constitutes the primary propellant of phantasy; without what Holmes (2020, p. 52), amongst others, describes as the “bottom-up” influence of core affective consciousness, “top-down” “psychic representation” (Ogden, 1984, p. 501) has no raison d'être. Accordingly, the shape of phantasy is driven both by the valence of affects that invoke it in the first place, as well as the “plots” around which psychic representation unfolds. Both these factors will be considered in turn.

Beginning with the valence of affect, the work of Wilfred Bion represents an important source of psychoanalytic insight, in particular his work on the “emotional link.” This concept involves the understanding that affect invariably constitutes an integral aspect of any object relation. In Bion's own words (Bion, 1962, pp. 42–43), “an emotional experience cannot be conceived of in isolation from a relationship. The basic relationships that I postulate are (1) X loves Y; (2) X hates Y; and (3) X knows Y.” These various emotional links would, in Bion's publications from the early 1960s, come to be referred to as (1) L, (2) H, and (3) K links. One of the important attributes of Bion's proposals were that, as Golse (2019) notes, they went someway “beyond the Freudian model” of the drives to incorporate the centrality of object relations within emotional experience. Furthermore, these ideas would have significant implications for Bion's understanding of the clinical encounter, as to comprehend the emotional link of any given session is, as Symington and Symington (1996, p. 29) write, to discover the “key” to the analytic hour “rather like the key signature at the commencement of a piece of music.”

This collection of Bionian ideas—particularly the notion that “emotional experience cannot be conceived of in isolation from a relationship”—is highly compatible with an understanding put forward earlier in this paper in respect of the basic emotion systems: principally that they are *relational drives*, the satisfaction of which routinely depends on objects (in the psychoanalytic sense) (Solms, 2018, p. 5). In short, within both the Bionian and the neuropsychoanalytic frame of reference, emotion is understood to be the *essential catalyst* of any object relation. Moreover, the basic emotion systems that Panksepp described can arguably add a retrospective layer of definition to Bion's theory insofar as these frameworks map onto each other with remarkable congruence. Without wishing to reduce the specificity of each of these models, three of the basic emotions (LUST, CARE, PLAY) might be more broadly considered L links; three (RAGE, FEAR, PANIC/GRIEF) could be conceived of as H links; whilst SEEKING bears a striking resemblance to the K link.^3^ Bringing together the language of both frameworks, the valence of affect can be said to play the fundamental role of infusing psychic representation with an emotional colour that propels (in ways that are highly specific to the basic need being activated) the unfolding link between “X” and “Y.” What's more, from the clinical standpoint, the fundamental question that the neuropsychoanalytic model developed by Solms (2018) urges therapists to ask—namely *what is the patient feeling?*—may reveal sessional keys that harbour the potential to facilitate a similar level of psychic access to that which Bion envisioned was enabled through identifying emotional links, as he conceived of them. Finally, drawing on the neuroanatomical exploration detailed in the sections above, it can be reasonably hypothesised that the PAG is likely to play an important part in any such emotional (and therein intrinsically relational) experience such as that described.

Turning now to the nature of the top-down plots that affect fuses into, the writing of Kernberg (1973, p. 364) (which, in turn, draws heavily on Ronald Fairbairn's work) is pertinent to the present discussion; specifically, the conceptualisation of “self-object-affect units.” In Kernberg's view, such units are a product of the ego's “primary autonomous functions”—which are said to include perceptual capacities—as well as the capacity to “introject experiential engrams and dichotomize them according to valence, positive or negative” (Robbins, 1980, p. 480). Crucially, these introjected units are described as comprising “the ‘building blocks' of the psychic apparatus, *which is at first a dual structure*” (Robbins, 1980, p. 480, my emphasis). For the purposes of this discussion, the notion that the ego functions to inculcate psychological foundations that are “*always already*” (Heidegger, 1953, p. 75) of a dual structure (and that it is doing so “at birth”) (Robbins, 1980, p. 480), is of clear relevance. Moreover, it can be seen to describe something fundamental of phantasy: that the psychic representation of affect unfolds around dyadic plots that are intrinsically structured as self-object networks (or “X-Y” configurations in Bion's aforementioned algebra).

Appropriating Sullivan's (1896, p. 5) architectural aphorism that “*form ever follows function*,” what, we might ask, does the dyadic format of phantasy reveal about its function? To consider this, the notion that the basic emotion systems represent relational drives is once again worth re-stating and under-scoring (Solms, 2018, p. 5). As a result of the basic biological and relational requirements demanded by these systems, being equipped with what Hopkins (2016, p. 2) has evocatively referred to as an “innate virtual reality generator” in phantasy confers clear evolutionary advantage. In the language of Friston (2012, p. 248), it is within this “virtual reality” that we “generate predictions” about how the self (and the body that the self is felt to reside within) might go about satisfying its needs in an environment populated with objects. As might be expected of any effective “predictive model” (Hopkins, 2016, p. 2), the structure of phantasy thus mirrors the very format of the world that it is functionally required to predict. Accordingly, the function of phantasy might be most readily discerned in its actualised effects on the world that it predictively relates to; as Golse (2019) observes, the relations we exhibit constitute external expressions of the affectively impelled internal links that connect the “virtual” self and object in phantasy.

A question that remains relates to the unconscious status that Kleinians ascribe to phantasy, as it could be argued that what is described above frequently goes on *within awareness*. If, however, the ego is, as Solms and Panksepp suggest, “unconscious in itself” then arguably its “primary autonomous functions” (such as the workings of phantasy) may most appropriately be described in consonant terms as “unconscious” phenomena. Moreover, returning to an idea expressed in the discussion of the second question above—that the new-born infant's object-relating occurs on a non-declarative level—then, given the influence that psychoanalysis understands our earliest experiences to have on our unconscious minds, the non-declarative level of “top-down” ego function may represent a particularly germane platform for conceptualising the primary location of unconscious phantasy. This suggestion is, in fact, closely aligned with a conclusion that Solms (2017, p. 94) draws; as he writes in his paper *What is “the unconscious,” and where is it located in the brain?*, Freud's system unconscious may be localised “in the non-declarative memory systems located beneath the cortex, primarily in the basal ganglia and cerebellum.” In consequence, whilst the id may no longer be a psychic agency with which unconscious phantasy can plausibly be associated—as, for instance, Ogden (1984, p. 501) explicitly does—the ego (primarily the non-declarative domain of ego functioning) does present a neuropsychoanalytically viable candidate in this respect.

## Conclusion

Whilst the arguments that split the British Psychoanalytical Society in the 1940s remain, for many in the psychoanalytic community, unresolved, adopting an interdisciplinary approach and drawing on developments from neighbouring fields can catalyse the generation of fresh perspectives. In particular, the work of Panksepp and Solms, as well as of others from the affective and cognitive neurosciences, can be fruitfully brought to bear on these controversial matters. As has been argued here, doing so can reveal renewed justification for aspects of the Kleinian metapsychology which regards the rudimentary ego, unconscious phantasy, and the primitive processing of affect via self-object networks, as intimately linked and ultimately fostered from the very beginning of life.

## Data Availability Statement

As a conceptual analysis article, this study did not generate any new data.

## Author Contributions

The author confirms being the sole contributor of this work and has approved it for publication.

## Conflict of Interest

The author declares that the research was conducted in the absence of any commercial or financial relationships that could be construed as a potential conflict of interest.

## Publisher's Note

All claims expressed in this article are solely those of the authors and do not necessarily represent those of their affiliated organizations, or those of the publisher, the editors and the reviewers. Any product that may be evaluated in this article, or claim that may be made by its manufacturer, is not guaranteed or endorsed by the publisher.

## Footnotes

^1^The name given to a BBC documentary covering the Brexit referendum, which saw the emergence of profound splits in British society. Appropriated in this instance in respect of the profound splits that emerged within the British Psychoanalytical Society in the 1940s.

^2^For the original charts produced to “illustrate the clustering process” (see Shallcross, 2014, p. 207; p. 208; p. 272).

^3^Bion would go on to describe “equal, but negative ties for each of the three links” (Golse, 2019). For example, -H is “*not to hate*”, which Stokoe (2020) has aptly described as the re-channelled aggression of “etiquette.” In a neuropsychoanalytic frame, these minus links might be conceptualised as “cognitive-affective amalgams” (Panksepp and Watt, 2011, p. 10): “top-down” modifications of the “bottom-up” basic emotions.
